# Supplementary material for: DMPK is a New Candidate Mediator of Tumor Suppressor p53-Dependent Cell Death
Source: Molecules. 2019 Sep 1;24(17):3175. doi: 10.3390/molecules24173175 (PMC6749264; doi:10.3390/molecules24173175)
Supplement: Supplementary file 1 [file molecules-24-03175-s001.pdf]

DMPK is a new candidate mediator of tumor suppressor p53-dependent apoptosis

Katsuhiko Itoh[1], Takahiro Ebata[1], Hiroaki Hirata[2], Takeru Torii[1], Wataru Sugimoto[1], Keigo Onodera[3], Wataru Nakajima[3], Ikuno Uehara[3], Daisuke Okuzaki[4], Shota Yamauchi[5], Yemima Budirahardja[1], Takahito Nishikata[1], Nobuyuki Tanaka[3], and Keiko Kawauchi[1, 3*]

Supplementary Information

Supplementary Materials and Methods

Reverse transcription-PCR (RT-PCR)

cDNA was prepared using PrimeScript 1st strand cDNA Synthesis kit (Takara). RT-PCR analysis was performed with PrimeSTAR HS (Takara) under the following conditions: 40 cycles of 98°C for 10 sec, 55°C for 5 sec, and 72°C for 10 sec. RT-PCR analysis of *DMPK* isoforms or *ACTB* as a control was carried out using the following primer pairs: mouse *DMPK-A* and *-B* isoforms forward 5´- ACTTCTCCAGCCAACTACAG -3´; mouse *DMPK-A* and *-B* isoforms reverse 5´- CCATCTAGATGGGAAGGTG -3´; mouse *DMPK-C* and *-D* isoforms forward 5´- ACTTCTCCAGCCAACTACAG -3´; mouse *DMPK-C* and *-D* isoforms reverse 5´- GCCATATGGGAAGGTGGATC -3´; mouse *DMPK-E* and *-F* isoforms forward 5´- ACTTCTCCAGCCAACTACAG -3´; mouse *DMPK-E* and *-F* isoforms reverse 5´- AGGCCTAGGGATCTGCGG -3´; mouse *ACTB* forward 5´-ATGGATGACGATATCGCTGCGC-3´; mouse *ACTB* reverse 5´-GCAGCACAGGGTGCTCCTCA-3´.

UV irradiation

Prior to irradiation, medium was removed. Subsequently, cells were irradiated with UVB or UBC by using the CL-1000 Ultraviolet Crosslinker (UVP, Upland, CA) or the CX-2000 Ultraviolet Crosslinker (UVP, Upland, CA), respectively.

Supplementary Figures

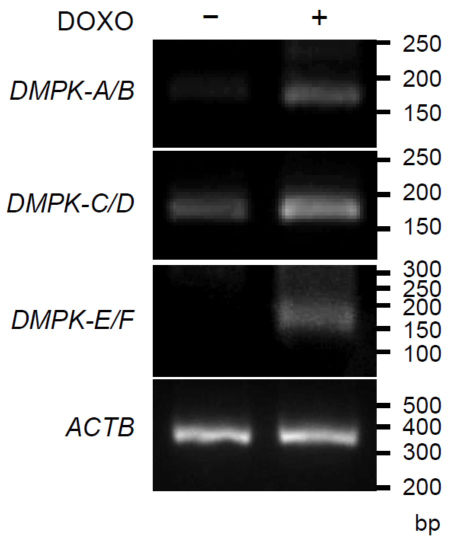

Figure S1. Identification of *DMPK* isoforms induced by DOXO treatment. *p53*$^{+/+}$ and *p53*$^{-/-}$ MEFs were treated with DOXO for 24 h. The expression of *DMPK* isoforms in untreated and DOXO-treated cells were evaluated by RT-PCR using specific primer sets for each isoform.

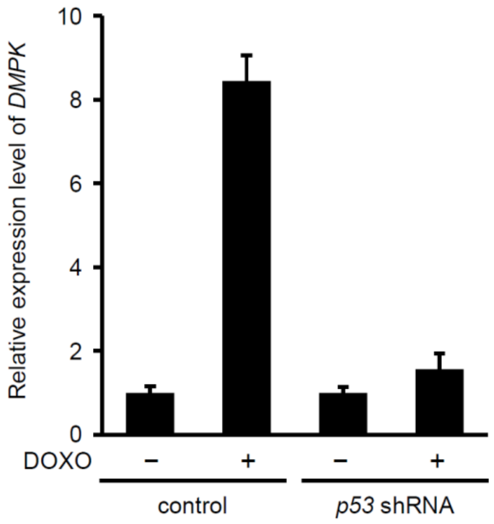

Figure S2. DOXO promotes *DMPK* expression in p53-dependent manner in MCF-7 cells. Control and *p53*-knockdown MCF-7 cells were treated with DOXO for 24 h. *DMPK* expression was evaluated by qRT-PCR. Each bar represents the mean ± SD; n = 3.

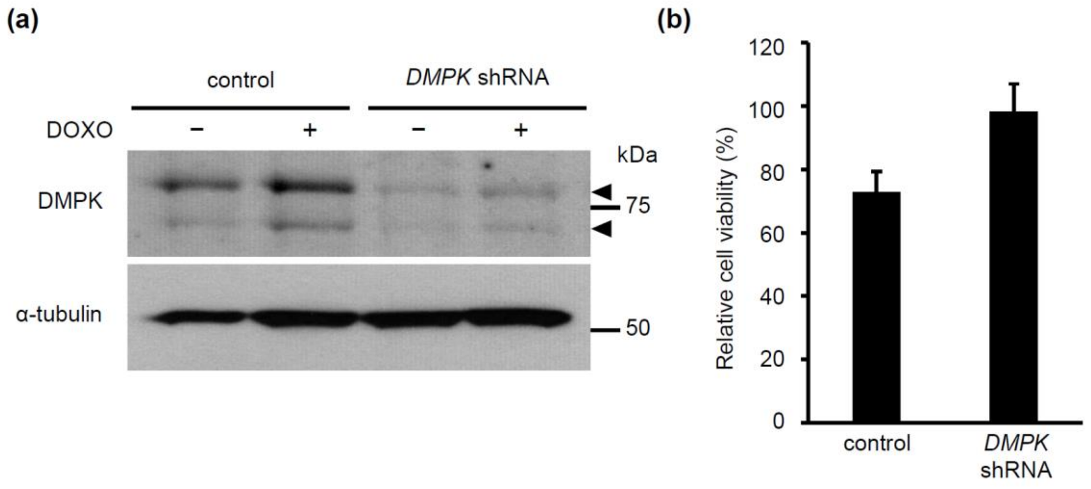

Figure S3. DOXO-induced growth inhibition of wild-type MEFs is attenuated by *DMPK* knockdown. WT (*p53*$^{+/+}$) MEFs infected either with control or *DMPK* shRNA-expressing retrovirus were treated with DOXO for 16 h. (a) Cell lysates were subjected to immunoblot analysis with antibodies against DMPK and α-tubulin as a loading control. (b) The cells were treated with DOXO for 24 h, and the number of viable cells was counted using trypan blue exclusion-based cell staining. In each condition, the number of doxorubicin-treated cells was normalized to that of non-treated cells. Each bar represents the mean ± standard deviation (SD); *n*=4.

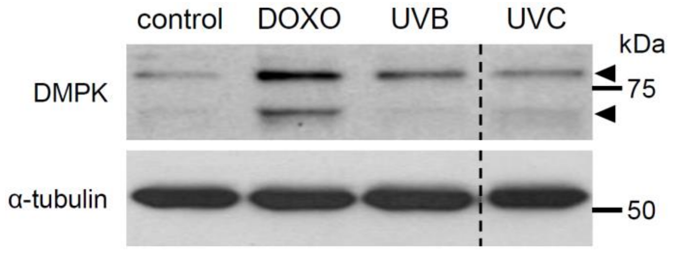

Figure S4. UV irradiation promotes DMPK expression. C2C12 cells were incubated for 16 h after UVB (1000 J/m^2) or UVC (10 J/m^2) exposure. Subsequently, cell lysates were subjected to immunoblot analysis with antibodies against DMPK and α-tubulin as a loading control.

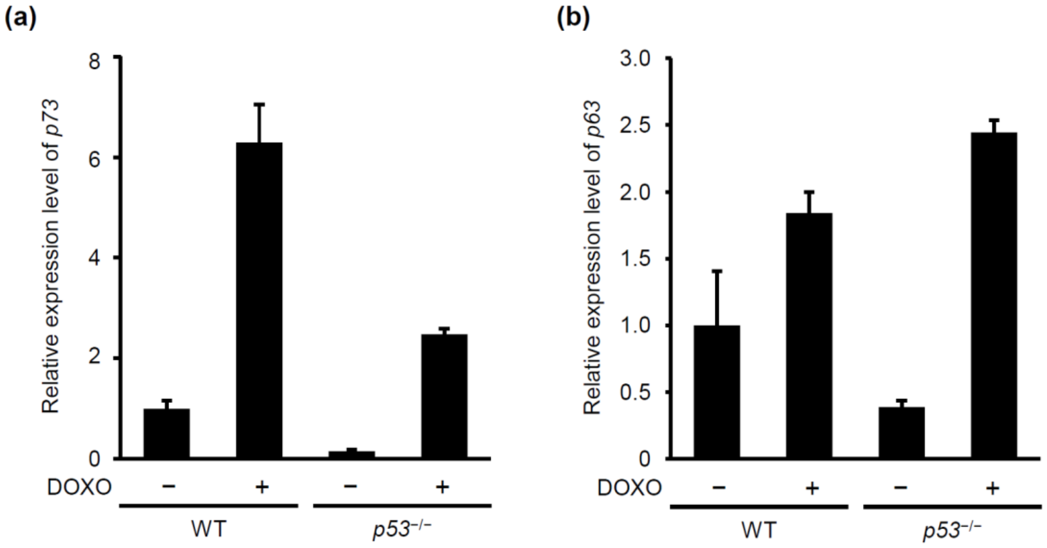

Figure S5. p53 deficiency does not influence DOXO-induced *p63* expression. Expression of *p73* (a) and *p63* (b) in *p53*$^{+/+}$ (WT) and *p53*$^{-/-}$ MEFs treated with or without DOXO for 24 h was evaluated by qRT-PCR. Each bar represents the mean ± SD; n = 3.
